# How do existing HRSN assessment tools assess social needs for older adults? A systematic review

**DOI:** 10.3389/frhs.2026.1952389

**Published:** 2026-09-23

**Authors:** Scott E. Wilks, Laura Ainsworth, R. J. Bardales, Julia Trahan

**Affiliations:** 1 Louisiana State University Healthy Aging Research Center, Baton Rouge, LA, United States; 2 Louisiana-Mississippi Geriatric Workforce Enhancement Program, Baton Rouge, LA, United States; 3 Louisiana State University School of Social Work, Baton Rouge, LA, United States

**Keywords:** geriatric, HRSN, older adults, outcomes measures, reliability, systematic review, validity

## Abstract

**Introduction:**

Health related social needs (HRSNs) limit access to healthcare and contribute to significant health disparities among older adults. The Louisiana-Mississippi Geriatrics Workforce Enhancement Program (LAMS-GWEP) is a federally funded program that educates and trains healthcare professionals to address the needs of older adults living with Alzheimer's disease and related dementias (ADRD) and their care partners. Given that HRSNs compound caregiving stress and may limit access to dementia-related healthcare, understanding how to accurately assess these needs in geriatric context is essential to the aims of LAMS-GWEP. Accordingly, we (the LAMS-GWEP team) conducted a systematic review with a threefold purpose: (a) to identify studies that applied an HRSN-focused empirical measure with older adult samples; (b) to assess the methodological rigor of these studies; and (c) to examine psychometric properties and utility from the HRSN assessment tools among these studies.

**Methods:**

Using the PRISMA methodology, inclusion criteria included publication within 10 years; use of an HRSN focused assessment tool; and geriatric samples in the healthcare setting. Exclusion criteria included gray literature, or publications not controlled by traditional commercial publishing; and psychiatric care settings. Each study was assessed for methodological rigor using the Newcastle-Ottawa Scale (NOS) adaptation for cross-sectional analysis. The studies were reviewed for findings of their respective HRSN psychometric properties: reliability, validity, cultural adaptability, acceptability, feasibility, and responsiveness.

**Results:**

Seven studies (*N* = 7) met inclusion and exclusion criteria. Methodological rigor of the studies' designs was high, and the sample of studies mentioned six HRSN assessment tools. Regarding psychometric properties and utility, none of the seven studies reported any statistics related to reliability. Two studies reported at least one type of validity with the geriatric population.

**Discussion:**

Literature that assesses geriatric-related social needs using HRSN assessment tools is scarce. Further, the tools used in this limited body of knowledge overwhelmingly lack adequate psychometric testing and understanding. Implications from this review address the need for formal testing of these tools with older adult samples to ensure empirically accurate representation of HRSNs within geriatric communities. This enhanced understanding should increase age-friendly initiatives endorsed by Centers for Medicare and Medicaid services and other geriatrics organizations.

## 1 Introduction

The older adult population in the United States is the fastest growing age group, with growth reported most recently by Wilder and Mackun (1) of 9.4%. This growth requires healthcare agencies and organizations to adapt to better serve older adults. To answer the growing social needs of older adults, assessment tools were developed for health related social needs (HRSNs) using previously developed assessment tools. By adapting them together on a new, condensed tool, it became easier to administer in the clinical setting (2).

### 1.1 Health related social needs

Health related social needs (HRSNs) are defined as concerns at an individual level that affect healthcare (3). Derived from social determinants of health (SDOH), they differ from SDOHs because SDOHs are community-level issues and HRSNs are individual-level issues (3, 4). While many HRSNs have been identified, the Centers for Medicare and Medicaid Services (CMS) have identified the following core HRSNs for inpatient and outpatient healthcare facilities to assess and monitor: food, housing, transportation, utilities, and interpersonal safety (5). Thus, these will be the focus of the present study to assist healthcare and other agencies assessing the needs of older adults.

Social needs relating to food are defined as concerns about accessing food financially, socially, or physically (6). Prevalence of food insecurity among adults over 50 is 13.1% (7). Older adults may have different presentations of food insecurity than younger counterparts, such as difficulty in preparing it (8). Sassine et al. (9) developed an assessment tool for food insecurity specific for use with older adults that captures physical burdens that may limit food access, the 6-item Physical Food Security tool. However, it does not include the financial aspects of food insecurity.

Housing problems involve transient housing, unaffordability of housing, unsafe neighborhood, and quality of housing and affect up to 15% of older adults (10). Differences in housing problems in the older adult population may include difficulty with the physical lay-out of the house or ease of accessing essential areas of the household, which may not be captured on routine housing problem assessment tools. Assessment tools do exist that have been used in older adult populations for housing problems [i.e. (11),], but they have not been validated. Lee et al. (12) was able to validate certain questions on a survey for older adults' functioning in relation to their housing arrangements, but it did not include housing quality, affordability, or security.

Transportation is defined as movement from one location to a destination, usually in a planned way (13). Transportation problems are anything that would be a barrier to that action. Prevalence of transportation problems among older adults is about 14% (14). Specific concerns given by older adults on the use of public transportation include its poor reliability and poor attitudes the transportation staff may have toward older adults (15). These are not always reflected in the most common questions about transportation problems. Furthermore, for the assessment tools that include questions relating to transportation problems (16), they have not yet been validated specifically for the older adult population.

Social needs relating to utilities usually involve trouble paying any bills, such as the electricity bill or water bill. Prevalence of trouble paying bills for older adults is 18% (14). Medical conditions faced by some older adults, such as cognitive impairment, may limit bill payment capabilities. Older adults with ADRD are up to eight times more likely to need bill payment assistance (17). Although validated assessment tools exist for older adults assessing capacity for handling finances (18) and potentiality for financial abuse (19), no validation study could be found to the best of the authors' knowledge for general bill payment hardship specifically for the older adult population.

Social needs relating to safety include any risk to safety in a person's living environment (20). Prevalence of safety concerns depends on how safety is measured, but previous research on safety among older adults has focused on neighborhood and environmental safety, with up to one-quarter of older adults feeling unsafe (21). Elder abuse is estimated to occur at a rate of 10% (22) and includes hitting, threatening, or other acts of violence, and financial exploitation (23). For this reason, safety concerns may be broader in definition for older adults. Specific assessment tools for elder abuse have been developed and validated (19), but there appears to be lacking interpersonal or neighborhood safety screening tools specifically validated for use on older adults.

### 1.2 Louisiana-Mississippi geriatrics workforce enhancement program

The Louisiana-Mississippi Geriatrics Workforce Enhancement Program (LAMS-GWEP) is a joint-state program funded by a federal grant to educate and train geriatric healthcare professionals, including physicians, nurses, and social workers (24). The education and training are focused on addressing the needs of older adults living with ADRD and their care partners (24). Older adults with ADRD number over 6 million currently and the number is expected to double in the next 30 years (25).

HRSNs affect individuals with ADRD in ways they may not affect others without ADRD. Executive functioning, a skillset affected by ADRD (26), is negatively associated with food insecurity in that as executive functioning declines, food insecurity increases (7). Transportation problems affect not only access to medical appointments or social events but also access to specialized day programs that help ease the burden of care (27). With elder abuse being a heightened concern for older adults with ADRD, safety and housing problems should be carefully considered and assessed. Problems with utility and bill payments may not always be financially based but may also be related to memory impairment. Gresenz et al. (28) found a rise in bill payment delinquency occurs in the years prior to an ADRD diagnosis, with this delinquency probability improving after diagnosis most likely due to family and care partner intervention.

While accurately assessing HRSN among older adults with ADRD and their care partners is important for the reasons described above, the LAMS-GWEP team did not find, through preliminary literature searches, that enough research has been done with this population to inform a systematic review for this population alone. Therefore, we modified the scope of this review to include community dwelling older adults which will also capture any study populations of older adults with ADRD and their care partners.

### 1.3 Purpose and research question

Older adults are at risk for communication differences in the healthcare setting. Vamos et al. (29) discussed barriers in healthcare communication faced by older adults including provider time limits with patients, poor understanding of health literacy level of patient by the providers, language barriers, and cultural differences. Outcome measures should meet communication needs by both older adults and their providers. When outcomes measures are designed, their target population should be considered, and validity should be obtained with that target group (30).

This systematic review of literature on HRSN assessment tools holds a threefold purpose: (a) to identify recent studies that applied a specific HRSN-focused assessment tools with older adult samples; (b) to assess the methodological rigor of these studies; and (c) to examine psychometric properties and utility from the HRSN assessment tools among these studies. The research question driving the search adhered to the PICOS framework as follows: For older adults (P), how are HRSN assessment tools [I (C-none)] used and reported on for the operationalization of social needs (O), based on quantitative, mixed type, or qualitative with quantitizing data study designs (S)?

## 2 Methods

For this review, we searched these comprehensive databases: Academic Search Complete, PsycINFO, CINAHL Complete, MEDLINE, and SocINDEX with Full Text. Each database was searched through the EBSCOhost platform. The Preferred Reporting Items for Systematic Reviews and Meta-analyses (PRISMA) and associated checklist were used to guide the search and procedures (31). The search concluded on March 29, 2026.

### 2.1 Inclusion and exclusion criteria

Using the PICOS framework and research questions as guides, the inclusion criteria were established as the following: the sample must be community-dwelling older adults with specified or average age of 60+; an inpatient or outpatient healthcare facility setting, including but not limited to emergency rooms, physician offices, or hospitals; an explicitly named empirical scale focusing on HRSN assessment of more than one core HRSN; a quantitative, mixed type, or qualitative with quantitizing data study design; and publication in peer-reviewed journals. Core HRSNs were defined as food insecurity, housing problems, transportation problems, problems with utility bill payment, and safety concerns (32). Exclusion criteria were: gray literature, or publications not controlled by traditional commercial publishing; and study locations in psychiatric care facilities. Although it is recognized that some gray literature provides value to the body of research knowledge, it was excluded because the goal was to understand rigor of studies assessing HRSNs with older adults and the psychometric properties reported in them. Peer-reviewed studies use standardized reporting styles that help ensure studies include these characteristics sought in this review. Finally, the following parameters were applied to the search criteria: academic journals and published between 2016 and 2026, which covers a 10-year time frame similar to a past, older systematic review of health-related social conditions (33). Although the English language was not applied or used as a restriction, the utilization of English words in the search limited the results to journals with English language keywords, thus inadvertently reducing results in other languages.

### 2.2 Search terms and selection process

The search utilized a Boolean technique for HRSNs, assessment, and the desired setting. To focus on social needs related only to healthcare within the conceptualization defined by CMS (3), the terms for HRSNs were “*health-related social needs” OR “health related social needs OR “HRSN” OR “HRSNs”*; to keep a broad search block allowing for all types of assessment tools, the terms for assessment were assess* OR eval* OR determ*; and to include healthcare settings, the terms for the desired setting were hospital* OR “acute setting” OR inpatient OR ward OR observation OR admission OR emergency OR ER OR ED OR outpatient. Specifications for other inclusionary and exclusionary criteria were ruled out during the review process to ensure broader, initial search criteria.

The search was carried out by one researcher, while two additional researchers conferred and agreed upon the search criteria, rules, and methods. The two additional researchers reviewed the final included studies to ensure consistency with agreed upon criteria, rules, and methods. All articles found in the initial search for each database were saved to a *new project* using the EBSCOhost platform and duplicates were removed. The reference titles were screened, and some were excluded based on the title. All other records were reviewed individually by abstract and then by full article as necessary, with any articles not readily accessible sought for retrieval through the interlibrary loan system or by contacting a librarian. All records sought for retrieval were retrieved. The records were excluded in the following order: study sample's average or specified age range below 60 years; study was not quantitative or mixed type study design; or the study did not use a named, HRSN-focused assessment tool. If a record was primarily in another language without a full English translation, an online translation service was utilized, Google Translate, to assist in translation of the record to determine eligibility. Once records were identified as meeting eligibility criteria, they were selected for data collection and analysis. Figure 1 shows the PRISMA flowsheet (31) for the search process.

**Figure 1:**
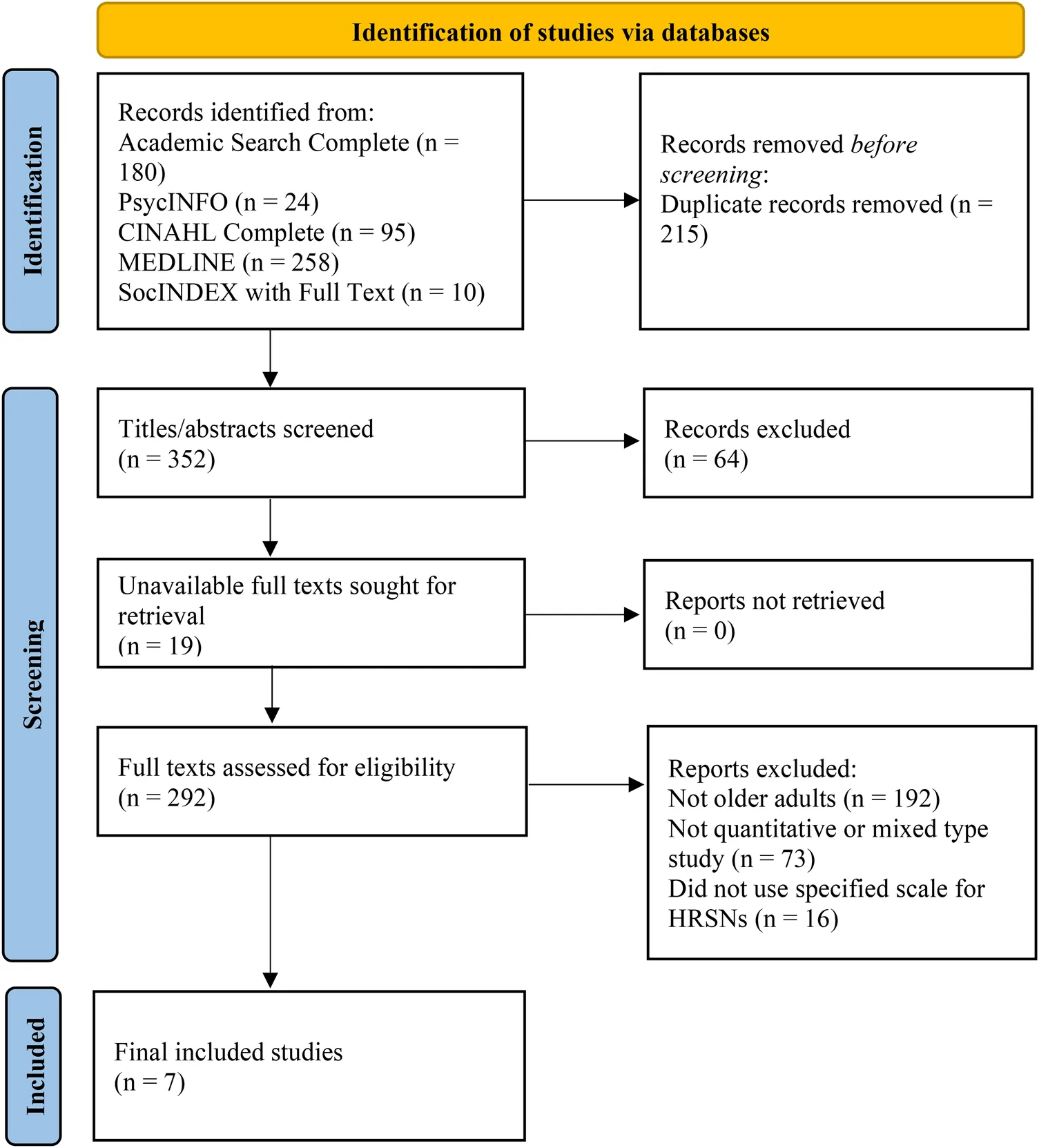
PRISMA flowsheet for systematic review. Citation and source: (31), License: CC BY 4.0 per https://www.prisma-statement.org/citing-prisma-2020, adapted.

### 2.3 Analytic approach

Data collection for each eligible study included each study's sample characteristics, type of study, purpose, methods utilized, scale used for HRSN assessment, and outcome. The data were tabulated and findings were synthesized by two researchers. We examined the methodological rigor and outcomes of the studies and identified the specific assessment tools used by each study. The varying assessment tools were then tabulated separately, and findings were synthesized by two authors.

#### 2.3.1 Assessment of methodological rigor

The Newcastle-Ottawa Scale (NOS) was originally built for case control or cohort studies, with a specific version for each (34). It was also adapted for cross-sectional studies by Herzog and colleagues (35) for use in assessing quality of methodology in a healthcare-related systematic review. The NOS was originally developed for use in systematic reviews to assess the quality of studies that have nonrandomized samples (34). A study evaluating the cross-sectional adaptation of the NOS against another tool, the Appraisal Tool for Cross-Sectional Studies (AXIS), found no clear advantage for the use of one over the other (36); therefore, the adaptation for cross-sectional studies was used for this systematic review. This decision was made after the search was completed and it was discovered that they were all cross-sectional designs and in healthcare settings, for which the NOS adaptation was designed (35). The NOS for cohort studies and the cross-sectional adaptation have three sections, *Selection*, *Comparability*, and *Outcome*. For cohort studies, Selection has four items with the possibility of four stars awarded for representativeness, selection of non-exposed cohort, gathering of exposure, and indication that outcome was not present at the beginning of study. For cross-sectional studies, Selection has four items for rating a study with a possibility of five stars applied for representation of the sample, size justification of the sample, discussion of comparison between non-respondents and respondents, and use of validated measurement tools. For both cohort and cross-sectional studies, Comparability has one item with the possibility of two stars to be applied for the study controlling for their primary factor and at least one other factor. For cohort studies, Outcome has three items with the possibility of three stars given for assessment of outcome, follow-up, and adequacy of follow-up reported. For cross-sectional studies, Outcome has two items and a possible three stars to be applied for the study in how the outcome was assessed and statistically tested. The NOS for cohort studies has a range of 0–9 and the NOS adaptation for cross-sectional studies has a range of 0–10 stars. Two authors rated each study independently and conferred after both time intervals to agree upon final rating for each study. The kappa statistic was calculated with STATA SE 17.0 to assess inter-rater reliability.

#### 2.3.2 Assessment of HRSN scale properties

Each assessment tool used to measure HRSNs in the studies was identified, and any original assessment-building study or referenced work was retrieved. The study included in this review, the original work for the assessment tool used in the study, and any other assessment-building studies found for that assessment tool were all used to tabulate findings about the used assessment tools included in the review. Characteristics of the tools were tabulated, including the name of assessment tool (with number of items), any other tools from which some portions of the used assessment tool were derived, HRSN domains covered by tool, and psychometric properties reported by included studies. The psychometric properties of the assessment tools were analyzed similar to another systematic review by Mansfield et al. (37), in which six areas were outlined for analysis of psychometric properties: reliability, validity, cross-cultural validity, responsiveness, acceptability, and feasibility. Reliability was assessed by a report of one of the following within the included studies: internal consistency, test/retest, interrater reliability, or generalizable coefficient (38). Validity was assessed by whether the assessment tool had face, content, criterion, or construct validity (37). Cross-cultural validity was assessed by whether the tool's psychometric properties were tested after use in another language or cultural group to ensure they are similar to the tool's original psychometric properties (39). Additionally, cross-cultural validity assessment included whether the authors conducted confirmatory factor analysis, measurement invariance, or differential item functioning (40). Responsiveness was assessed by whether the assessment tool can detect change after passage of time with significance (39). Acceptability was assessed by whether the assessment tool was reportedly received well by respondents. Feasibility was assessed by whether the assessment tool was reportedly able to be administered efficiently by researchers (37).

## 3 Results

A total of 567 records were identified in the initial search. After duplicates were removed and record titles were screened for appropriateness of inclusion, 292 records were examined further for possible inclusion. Reports were excluded as per criteria, leaving seven records for final inclusion. Figure 1 details the process in the PRISMA flowsheet. The most common reason for non-inclusion was the sample's average age. The second most common reason for non-inclusion was the study was not a quantitative or mixed type study. The remaining excluded articles were due to the study not using a specified scale for HRSNs. The final seven articles were authored by Chary et al. (41), Kaminski et al. (42), Khemraj et al. (43), O'Leary et al. (44), Russell et al. (45), Schultz et al. (46), and Wright et al. (47).

All studies included were published within the last three years of this review. Mean sample ages ranged from 60.1 to 80.1 years and sample sizes of older adults ranged from 68 to 450. Settings ranged from outpatient clinics, specialty services, and inpatient services. A summary of each study's sample characteristics and setting, purpose, methods, HRSN assessment tool used, and outcomes is presented in Table 1.

**Table 1:** Summary of included studies.

Authors	Sample characteristics, design, and setting	Purpose of study	Methods utilized	HRSN scale utilized, core HRSNs assessed	Outcome(s), age stratification	NOS adaptation score
Chary et al. (41)	Patients having visited the ED 4x+ in one year Black = 40%; White = 28%; Mean age = 67 (range = 57–82 years) *n* = 68 Design: Cross-sectional Setting: ED	Identify reasons of frequent use of ED among older adults	In-person interview once during ED visit; content analysis of qualitative portion	Emergency department adaptation of the AHC HRSN screening tool to assess food insecurity, housing problems, transportation problems, and utility concerns.	Housing concerns = 32%; Transportation concerns = 23%; Food concerns = 29%; Utility concerns = 6%; Six profiles were identified, relating to insurance concerns, concerns about ongoing illnesses or diseases, and acute medical concerns Results not stratified by age	5
Kaminski et al. (42)	Adults aged 65 and over who had been hospitalized in the ICU Non-Hispanic White = 67.3%; Mean age = 80.1 (80 years or olde*r* = 52%) *n* = 450 Design: retrospective cohort design Setting: ICU	Understand prevalence of specific HRSNs among older adults who have experienced a critical illness	Review of NHATS survey answers and linkage to Medicare revenue files	NHATS survey portions for transportation and food insecurity	HRSNs increased within the year after ICU discharge Results stratified by age only for pre-ICU stay: 80 years and older made up 59% of those with food insecurity and 72.3% of those with transportation problems.	8
Khemraj et al. (43)	Adults with symptoms of depression with low income Black = 52%; White = 41%; Mean age = 70.8 (SD = 9.1) *n* = 132 Design: Cross-sectional Setting: Primary care clinics and community agencies	Understand association of HRSNs and depression	Participants received telephone assessments	AHC HRSN screening tool to assess food insecurity, housing problems, transportation problems, utility concerns, and safety	Food insecurity is correlated with increased depression Results not stratified by age	9
O'Leary et al. (44)	Hospitalized patients White America*n* = 60.5%; Mean age = 60.1 (+/- 16.7 years) *n* = 413 Design: Cross-sectional Setting: Hospital	Assess differences in hospital and research staff HRSN screening results and compare results from two different instruments	Patients were screened upon admission by healthcare staff and on second or third hospital day by research staff by interview	AHC HRSN screening tool to assess food insecurity, housing problems, and transportation problems	Hospital staff identified fewer HRSNs than research staff; The AHC HRSN screening tool and unnamed HRSN assessment tool were minimally different in outcome Results not stratified by age	7
Russell et al. (45)	Subgroup: Veterans at a suburban clinic Non-Hispanic Black = 6%; Non-Hispanic White = 81.7%; Hispanic = 4.1%; Mean age = 61 (age 65 or olde*r* = 54%) *n* = 184 Design: Cross-sectional Setting: Suburban clinic	Assess prevalence of HRSNs among veterans and test a new system for measuring and providing resources for HRSNs	Self-report screening survey given in waiting room during routine visit to VA clinic	ACORN screening tool to assess food insecurity, housing problems, transportation problems, utilities, and safety	42.4% reported at least one HRSN Results stratified by age in total sample, not just subgroup: Ages 65 and up = 17.9% reported at least one HRSN	8
Schultz et al. (46)	Patients living in low resource, high deprivation geographical areas with known or suspected cancer who are about to receive surgery for it White = 91%; Hispanic = 5.2%; Mean age = 66 (range = 57–73 years) *n* = 387 Design: Cross-sectional Setting: Surgical center	Understand psychosocial needs among patients with cancer receiving surgery for it	Survey administered by phone prior to the planned surgery	Socioecological Determinants of Health-88 adaptation to assess food insecurity, housing problems, transportation problems, utilities, and safety	Over half had at least two social needs Results not stratified by age	8
Wright et al. (47)	Patients in a dialysis clinic Black = 60.2%; White = 31.5%; Mean age = 62 (range = 22–84 years; *n* = 73) Design: Cross-sectional Setting: Dialysis clinic	Use Plan-Do-Study-Act to implement HRSN screening at a dialysis clinic	Self-administered surveys patients completed during clinic visit	Core 5 screening tool to assess food insecurity, housing problems, transportation problems, utilities, and safety	HRSN identification was improved at the clinic and the Core 5 was found to be useable by nursing staff sResults not stratified by age	6

HRSN, health related social needs; NOS, Newcastle-Ottawa scale; ED, emergency department; AHC, accountable health communities; ICU, intensive care unit; NHATS, National health and aging trends study; ACORN, assessing circumstances and offering resources for needs.

Khemraj et al. (43) and O'Leary et al. (44) utilized the CMS Accountable Health Communities (AHC) HRSN screening tool. Khemraj et al. (43) used baseline survey data from another study with participants from several settings including a primary care clinic (48) to understand the association between depression and the following HRSNs: housing problems, transportation problems, food insecurity, safety concerns, and utility problems. Food insecurity was the only HRSN significantly associated with depression (43). O'Leary et al. (44) analyzed differences of HRSN screening results administered by hospital staff and research staff, finding that hospital staff identified fewer HRSNs than research staff. The core HRSNs measured were the following: transportation, food insecurity, and housing. They discussed implications of their findings for hospitals to improve screening techniques.

Two studies used adaptations from other assessment tools. Chary et al. (41) used an adaptation of the AHC HRSN screening tool, adapted for use in ED (49), but they also used the Health Leads screening toolkit which recommends specific questions and alternative questions derived from many other screening tools for consideration. Chary et al. (41) did not explicitly describe how they used the toolkit to inform their use of the AHC HRSN screening tool. They utilized mixed methods to analyze a sample who had frequented the ED at least four times in a year, asking them about the following core HRSNs: housing, food insecurity, utilities, and transportation. Well over half of participants had at least one concern, but the qualitative portion of the study revealed participants did not attribute their HRSN concerns as contributing to their frequent ED usage (41). Schultz et al. (46) used an adaptation of the Socioecological Determinants of Health-88 screener. Schultz et al. (46) assessed HRSNs among patients scheduled to receive intent-to-cure surgery for known or suspected cancer. Their screener had 27 domains, 17 of which were social. Although they did not list every HRSN measured by the screener, the results indicated that nearly a third of respondents had at least one of the following core HRSN concerns: housing, food insecurity, utilities, transportation. The remaining core HRSN, safety, was also included in the screener but not reported in the study.

Kaminski et al. (42) used survey portions from the National Health and Aging Trends Study (NHATS) that contained validated measures for HRSNs, which will be discussed later in these results. They merged data from the NHATS with Medicare revenue files that indicated which participants had been hospitalized in an ICU setting. Using these data sets, they analyzed possible effects of an ICU stay on the following core HRSNs: transportation and food insecurity. They found prevalence of HRSNs increased after ICU stays (42).

Russell et al. (45) built a screening tool during an initiative, Assessing Circumstances and Offering Resources for Needs (ACORN), designed to improve screening and referrals for veterans with HRSNs. They examined two groups of veterans, one from an urban Veteran Affairs (VA) clinic and one from a suburban VA clinic. The group from the suburban clinic comprised primarily older adults with average age of 61 years and analyzed separately for comparison. They developed an HRSN screening tool and worked to validate its use for veterans. The core HRSNs included on the ACORN screening tool were the following: food insecurity, transportation, utilities, housing, and safety concerns. They found 42.3% of the suburban group was positive for at least one HRSN, with 9.2% having three or more needs (45).

Wright et al. (47) used the Core 5 screening tool for their study. They systematically incorporated HRSN screenings at a dialysis clinic using the Plan-Do-Study-Act (PDSA) process. They saw improvements in the HRSN screening process after implementing the PDSA. The Core 5 was found to be useful by staff and measured the following core HRSNs: transportation, housing, food insecurity, utilities, and safety (47).

### 3.1 Methodological rigor of studies

Six studies were cross-sectional and one study was a retrospective cohort study. The average score on the NOS adaptation for cross-sectional studies was 7.2, indicating overall high quality of the methodology of cross-sectional studies (50). The NOS adaptation for cross-sectional scores ranged from 5 to 9. None of the cross-sectional studies met the full Selection quality measures, four met the full Comparability quality measures (43–46), and two met the full Outcome quality measures (43, 45). The highest concern for bias among the cross-sectional studies arose when considering the comparable characteristics of respondents vs. non-respondents and satisfactory response rate, which is are items within Selection. Only one study met this requirement (46). The least concern for bias among cross-sectional studies was in the use of validated instruments for HRSNs, as each study reported or gave reference to an HRSN tool that has been given validity of some type. Table 2 displays the stars awarded each cross-sectional study for Selection, Comparability, and Outcome.

**Table 2:** Results of Newcastle-Ottawa scale adaptation for cross-sectional studies.

Authors	Selection (max = five stars)	Comparability (max = two stars)	Outcome (max = three stars)	Total (Max = 10)
Chary et al. (41)	* * * *		*	5
Khemraj et al. (43)	* * * *	* *	* * *	9
O'Leary et al. (44)	* * *	* *	* *	7
Russell et al. (45)	* * *	* *	* * *	8
Schultz et al. (46)	* * * *	* *	* *	8
Wright et al. (47)	* * * *		* *	6

The retrospective cohort study by Kaminski et al. (42) scored a 9 out of a possible 9, showing high methodological rigor. They had a representative sample, selected all participants from the same cohort, gathered data from a secure record, used demographic data and many other variables as controls, linked record to Medicare revenue files, analyzed follow-up data post-ICU stays, and detailed participant attrition. Types of assessment tools and their validity findings will be discussed in the next section. The interrater reliability statistic was *k* = 0.75, indicating moderate-to-strong reliability (51).

### 3.2 Psychometrics of the HRSN measures used in studies

Six different assessment tools were used in the current review's qualified studies: the ACORN screening tool, the AHC HRSN screening tool, the AHC HRSN screening tool adaptation for emergency departments, Core 5 screening tool, the NHATS survey, and the Socioecological Determinants of Health-88 screener adaptation. Two studies used the original AHC HRSN screening tool, and one other study used an adapted version of it. Two assessment tools, ACORN (45) and the adaptation of the Socioecological Determinants of Health-88 (46), were developed by the authors of the respective studies. For the other assessment tools, the referenced study was located and examined for methodology used in the design and any reported psychometric properties. The most frequent psychometric property reported was content validity. None of the included or referenced articles reported responsiveness of the HRSN assessment tools or any psychometric properties relating to their respective reliability. Some findings for the psychometric properties were not based on samples of older adults, which will be identified as each assessment tool is discussed. Table 3 presents used scale details and psychometric properties reported for each.

**Table 3:** HRSN assessment tools used by included studies.

Assessment tool description	Validity reports	Reliability reports	Feasibility and acceptability
ACORN screening tool (45) Core HRSN covered: Food, housing, transportation, utilities, and safety Original population for which developed: Veterans of all ages 14 items	Content: Yes, with subject matter experts Criterion: Not reported in included study Construct: Not reported in included study Cross-cultural: Not relevant to included study	Internal consistence: Not reported by included study Test/retest: Not reported by included study Interrater reliability: Not reported by included study Generalizable coefficient: Not reported by included study	Acceptability, determined by a small group of Veterans, age not specified (45)
AHC HRSN screening tool (43, 44) Core HRSN covered: Food, housing, transportation, utilities, and safety Original population for which developed: Any age and setting 10 items	Content: Yes, with expert panel (5) Criterion: Not reported in included or original studies (5) Construct: Not reported in included or original studies Cross-cultural: Not relevant to included studies	Internal consistence: Not reported by included studies Test/retest: Not reported by included studies Interrater reliability: Not reported by included studies Generalizable coefficient: Not reported by included studies	None reported by included or referenced studies
AHC HRSN screening tool adaptation for emergency department (41) Core HRSN covered: Food, housing, transportation, utilities, and safety Original population for which developed: patients visiting an emergency department 16 items	Content: Yes, with expert panel (5) Criterion: Not reported in included study Construct: Not reported in included study Cross-cultural: Translated to Spanish but no psychometric testing reported	Internal consistence: Not reported by included study Test/retest: Not reported by included study Interrater reliability: Not reported by included study Generalizable coefficient: Not reported by included study	None reported by included or referenced studies
Core 5 (47) Core HRSN covered: Food, housing, transportation, utilities, and safety Original population for which developed: adults at a healthcare clinic (2) 5 items	Content: Yes, with nurse experts Criterion: Not reported in included study Construct: Not reported in included study Cross-cultural: Not relevant to included study Other: Face validity through literature review	Internal consistence: Not reported by included study Test/retest: Not reported by included study Interrater reliability: Not reported by included study Generalizable coefficient: Not reported by included study	Feasibility reported by Bechtel et al. (2) with adults of various ages Acceptability reported by Wright et al. (47) as indicated by clinic staff
NHATS survey questions (42) Core HRSN covered: Food and transportation Original population for which developed: Adults 65 years of age and older who with Medicare 15 items	Content: Not reported in included or referenced studies Criterion: Not reported in included or referenced studies Construct: Yes (62) Cross-cultural: Not relevant to included study	Internal consistence: Not reported by included study Test/retest: Not reported by included study Interrater reliability: Not reported by included study Generalizable coefficient: Not reported by included study	None reported by included or referenced studies
Socioecological Determinants of Health-88 adaptation (46) Core HRSN covered: Food, housing, transportation, utilities, and safety Original population for which developed: Adult patients preparing for major surgery 136 items	Content: Yes, expert review Criterion: Not reported in included study Construct: Convergent validity indicated as supported due to results of screener being associated with known deprivation areas, but no statistics reported Cross-cultural: Not relevant to included study Other: Face validity reported through pilot testing (46)	Internal consistence: Not reported by included study Test/retest: Not reported by included study Interrater reliability: Not reported by included study Generalizable coefficient: Not reported by included study	None reported by included or referenced studies

HRSNs, health related social needs; ACORN, assessing circumstances and offering resources for needs; VA, veteran affairs; AHC, accountable health communities; HES, household energy security; PRAPARE, protocol for responding to and assessing patients' assets, risks, and experiences; HITS, hurts, insults, threatens, and screams.

#### 3.2.1 ACORN screening tool

The ACORN screening tool was developed by Russell et al. (45) for use in military veteran clinics and to improve the HRSN screening process used by those clinics. They used subject matter experts to review the screening tool and tested acceptability with a small group of Veterans (age not stated) prior to implementing with a larger sample of older Veterans. A reported limitation (45) was the risk of not being representative of larger groups of veterans in determining its acceptability.

Seven previous assessment tools were consulted and incorporated into the ACORN screening tool:

- *Veterans' Clinical Reminder for Food Scarcity*, a 1-item assessment of food insecurity with reported feasibility and acceptability (sample not older adults) (52);
- *Homelessness Screening Clinical Reminder*, a 2-item screening flow that assesses housing instability (53);
- *AHC HRSN Screening Tool,* a 10-item screening tool that assesses each CMS-targeted HRSN with reported content validity (sample not older adults) (5, 32);
- *Household Energy Security* (HES) indicator, a 1-item screener for ability to access and afford basic, necessary energy services with reported face validity (sample not older adults) (54);
- *WellRx*, an 11-item tool to assess barriers to non-medical, social determinants of health with reported feasibility (age not stated) (55);
- *Protocol for Responding to and Assessing Patients' Assets, Risks, and Experiences* (PRAPARE), a 20-item measure that matches patients with targeted community resources based on social drivers of health with reported content validity (sample not older adults) (56), construct validity and reliability (sample not older adults) (57), and feasibility (older adult sample) (58);
- *Hurts, Insults, Threatens, and Screams* (HITS)*,* a 4-item scale that assesses safety with reported internal reliability and criterion/convergent validity (sample not older adults) (59).

#### 3.2.2 Original and adapted AHC HRSN screening tool

The AHC HRSN screening tool was originally designed by CMS and detailed by Billioux et al. (5). The measure is used in healthcare clinics for people of any age and diverse backgrounds (5). CMS conducted an extensive search of literature, incorporated five previous HRSN screening tools, and had an expert panel to inform their process. Limitations of the CMS ACH screening tool as indicated by Billioux et al. are that it is not comprehensive for all HRSNs, lacks ability to narrow the need more specifically, and no reported testing for reliability (5).

Chary et al. (41) adapted the AHC HRSN screening tool for use in English and Spanish for cultural adaptability in hospital emergency department settings, but no testing was reported to show cross-cultural validity. Reported limitations of the tool are that it is limited to only two languages currently, and a lack of evidence for criterion validity (49).

PRAPARE, the HES indicator, and HITS were consulted and incorporated into the AHC HRSN Screening Tool as well as the following two tools:

- *Unnamed housing screener,* a 2-item questionnaire assessing housing conditions with reported content validity (sample not older adults) (60);
- *The Hunger Vital Sign*, s 2-item screener assessing food insecurity with reported convergent validity (tested against known factors relating to food insecurity among caregivers of children, not older adults) (61).

#### 3.2.3 The core 5

The Core 5 screening tool was developed for use among healthcare clinic adult patients who were middle-aged on average (2). A thorough literature review and a team of nurse experts informed its design, yielding face and content validity. The Core 5 was found to have feasibility by Bechtel et al. (sample not older adults) (2) and acceptability by Wright et al. (determined by sample of clinic staff, not older adults) (57). Limitations reported by Bechtel et al. (2) were primarily on the design of their study, with restricted generalizability being the main concern. Other reported limitations were possible social desirability bias and non-response bias in the measure's development. The AHC HRSN Screening Tool was consulted and incorporated into the building of the Core 5 screening tool.

#### 3.2.4 The NHATS survey

HRSN-focused items in the NHATS survey measures two of the CMS core HRSNs: food insecurity and transportation (42). Tucher et al. (62) suggested evidence of construct validity on the food insecurity items, specific for older adults. A reported limitation of the NHATS is that, in early development, it used only Medicare beneficiaries, limiting generalizability to those not receiving said benefits (62).

#### 3.2.5 Socioecological determinants of health-88 adaptation

Schultz et al. (46) designed a 136-item screener from the 88-item Socioecological Determinates of Health-88. Although Schultz et al. used the Socioecological Determinates of Health-88 screener as the basis for their adaptation, they sourced much of the scale from original reference articles and screening tools. During development, they reported the use of three reference screening tools: the Hunger Vital Sign, PRAPARE, and the AHC HRSN screening tool. Schultz et al. (46) reported evidence of face, content, and convergent validity in their sample of older adults. Limitations of the Socioecological Determinants of Health-88 include a possible lack of external validity due to lower response rates by marginalized communities (46).

## 4 Discussion

Overall, conspicuously few studies address older populations in healthcare settings when evaluating HRSNs. Accordingly, convincing evidence of the most applicable HRSN assessment tool for older adults is lacking. While the methodological rigor of the qualified studies in the current review was rather high, the chosen HRSN assessment tools for each study lacked disclosure of psychometric reliability within the included studies. Further, most of the reference assessment tools used to develop the aforesaid HRSN assessment tools lacked reported reliability within the referenced studies, save the PRAPARE (57) and HITS (59).

The research question driving the current systematic review asked how HRSN assessment tools operationalize social needs for older adults. Overall, the HRSN assessment tools used by the included studies have less than ideal reporting of reliability and validity psychometric properties (63), but five out of six assessment tools were comprehensive of all five CMS core HRSNs. The assessment tools were all carefully considered through their development and often relied on previously validated assessment tools in the referenced studies to help build the HRSN assessment tool used in the included studies. HRSN assessment tools rely heavily on face or content validity with few reporting any other form of validity or other psychometric properties. Health leads (64) encourages the use of measures that have, at minimum, construct validity and content validity. The Socioecological Determinates of Health-88 screener adaptation was the only measure in the current review that met this validity threshold (46).

Following Health Leads' (64) guidance, we caution against selecting assessment tools or items from assessment tools that were designed for specific populations that may not be generalizable to the person(s) in present need of HRSN assessment. The NHATS survey was designed for adults aged 65 years and older, but the included study used it for only two core HRSNs—food and transportation. The Socioecological Determinants of Health-88 adaptation was designed and used in Schultz et al.'s (46) study with a mean sample age of 66 years and showed two types of validity. However, their adaptation may not be feasible for routine inpatient or outpatient assessments due to its rather cumbersome length.

A popular checklist for prospective studies seeking to assess and test outcome measures recommends the prospective study select a sample that is representative of their target population (30). Using this checklist, only two measures (NHATS survey and Socioecological Determinates of Health-88 adaptation) would have confirmed validity specific to the current systematic review's target population, older adults. Reporting validity and reliability of measurement or assessment tools helps further research and improve future researchers and clinician in selecting the best tools to use (63). For only two assessment tools in this review to have validity with the geriatric population is glaring. The scales utilized in this review were not designed to capture the unique HRSNs faced by older adults. Although they should be considered by geriatric and gerontological researchers, neither the NHATS survey nor the Socioecological Determinants of Health-88 adaptation is ideal for use in a routine clinical practice. An obvious, future research direction would be to validate assessment tools that clinicians use for older adults.

The assessment tools utilized in the included studies did well in incorporating questions inclusive of the different ways HRSNs may affect older adults. Age-friendly food insecurity questions were demonstrated by two assessment tools, the ACORN screening tool and the NHATS survey. The ACORN screening tool asked if help was needed help getting food (45) and the NHATS survey asked if meals were missed because shopping or preparing food was too difficult or the individual did not have access to help (62). Age-friendly housing problem questions were demonstrated by the Socioecological Determinants of Health-88 screener by asking whether the individual felt physically and emotionally safe in their living environment (46). This can be interpreted as inclusive of internal or external safety concerns such as housing design problems that lead to falls. Age-friendly transportation problem questions were identified in the Core 5 screening tool, the AHC HRSN screening tool, and the Socioecological Determinants of Health-88 screener. They asked the same question, which was whether transportation problems kept the individual from medical appointments or other things needed for daily living (2, 5, 46). This question helps include concerns about ride reliability or cessation of driving. Age-friendly bill payment questions were seen in the AHC HRSN screening tool, Socioecological Determinants of Health-88 screener, and the ACORN screening tool. They all used the same question which was whether electric, gas, oil, or water companies had warned about services being turned off in the past 12 months (5, 45, 46). By not specifying it being due to financial reasons, this allows individuals to answer if that has happened to them due to them not being able to keep track of bills or physically pay them due to medical or cognitive concerns. Finally, age-friendly safety questions were identified in the AHC HRSN screening tool and ACORN screening tool. The AHC HRSN screening tool utilized four questions relating to safety and included forms of verbal, physical, and emotional types of abuse which are concerns some older adults may have (5). The ACORN screening tool safety question was worded differently but also was inclusive of all types of abuse from anyone (45).

Examination of not only the difference in manifestation of HRSNs among older adults but also the difference of measurement considerations that researchers and clinicians need to have when assessing older adults is a must to increase age-friendliness and comprehensiveness of care. Involving older adults as co-designers of a research study or in decisions relating to creating an age-friendly inpatient or outpatient heas ensure needs are understood and assessment tools are acceptable and feasible (65). Previous research using older adults as co-designers have used them in focus groups, interviews, discussions, brainstorming, and many other types of activities (65). A recent study by Norekval et al. (66) reviewed challenges when conducting research with older adults and ideas for addressing those concerns. Challenges related to sensory concerns such as vision or hearing impairment, physical concerns such as tiring easily when walking or writing, and cognitive impairment. Solutions presented included allowing time for older adults to complete forms and exams, offering accommodations that would alleviate physical burdens during tasks related to the research, reducing overstimulating external factors, using plain language, and allowing supportive assistance as needed. Norekval et al. (66) also recommended involving older adults in the planning stages of research studies to ensure methodologies and goals of the study support their concerns and interests. This current review did not find any studies that reported using an older adult sample to help inform their study design. Future research examining the HRSN concerns of older adults may consider bringing them in the early stages of the planned study.

This study is not without important limitations. First, limiting the search to the CMS core HRSNs may have other, important HRSNs that greatly impact older adult populations. Future direction would include further exploring other HRSNs that affect older adults and how they are measured and assessed in healthcare and mental health settings. Another limitation was having only one researcher engaged in the search may have led to selection bias. However, several pre- and post-review scoping searches completed at different time intervals revealed no other articles identified. Generalizability of findings may be limited due to using English search terms which may have inadvertently limited the inclusion of relevant studies in other languages. Additionally, although the five databases searched covers relevant journals and sources for this study, limiting the search boundaries to those five databases may have caused some relevant sources to be missed. Limiting the search to the last 10 years may have also left out earlier, relevant studies. Another limitation to this systematic review is the relative newness of healthcare agencies seeking appropriate HRSN assessments resulted in fewer studies. The small number of included studies may have weakened the overall strength of the conclusions.

Using existing psychometric properties with each identified assessment tool in the current review, it is difficult to draw conclusions on which scales would be fitting for healthcare practices among older adults. This is further complicated by the assessment tools being assessed only from the reports of the included studies. They may have validity or reliability reported elsewhere with other populations, but not with older adults. This does not indicate the assessment tool should not be used for older adults. Rather, it is a starting point for future research to increase in testing these assessment tools for the older adult population. The heterogeneity of the assessment tools, compiled of screening tools and surveys, also limits the comparison between them. However, we identified the psychometric gaps of these HRSN assessments, with the hope of encouraging future geriatric research that incorporates HRSN reliability and validity testing.

In summary, we identified studies that applied a specific HRSN-focused assessment tool with older adults, assessed those studies' methodological rigor and outcomes, and examined the properties and utility of said HRSN scales from those studies. Seven studies were identified and noted as holding sound methodological rigor, but the HRSN assessment tools had limited validity and no reported reliability within the included studies. This review highlights the disparity of outcome measures developed for older adults in the healthcare setting, a setting primarily used by older adult individuals and families.

## Data Availability

The original contributions presented in the study are included in the article/Supplementary Material, further inquiries can be directed to the corresponding author.
